# Supplementary material for: The Effects of n-3 PUFA Supplementation on Bone Metabolism Markers and Body Bone Mineral Density in Adults: A Systematic Review and Meta-Analysis of RCTs
Source: Nutrients. 2023 Jun 19;15(12):2806. doi: 10.3390/nu15122806 (PMC10303698; doi:10.3390/nu15122806)

Supplementary Content

p 1: Search strategy

PubMed: (n-3 pufa[Title/Abstract] or omega-3, fatty acids[Title/Abstract] or fatty acids, omega-3[Title/Abstract] or "Fatty Acids, Omega-3"[Mesh] or "Fatty Acids, Unsaturated"[Mesh] or unsaturated fatty acids[Title/Abstract]) and ("Osteoporosis"[Mesh] or "Bone Density"[Mesh] or osteoporosis[Title/Abstract] or bone health[Title/Abstract] or bone density[Title/Abstract] or bone mineral density[Title/Abstract] or "Bone and Bones"[Mesh] or "Fractures, Bone"[Mesh] or fractures, bone[Title/Abstract] or bones[Title/Abstract])

Web of Science: (TS=n-3 pufa OR TS=omega-3, fatty acids OR AB=n-3 pufa OR AB=omega-3, fatty acids) AND (TS=osteoporosis OR TS=bone density OR TS=bone mineral density OR TS=bone OR TS=bone loss OR AB=osteoporosis OR AB=bone density OR AB=bone mineral density OR AB=bone OR AB=bone loss)

EBSCO: (TI n-3 pufa OR AB n-3 pufa OR TI (omega-3 fatty acids or omega 3 or fish oil) OR AB (omega-3 fatty acids or omega 3 or fish oil) OR TI fatty acids, omega 3 OR AB fatty acids, omega 3 OR (MM "Fatty Acids, Omega-3+") OR TI fatty acids, unsaturated OR AB fatty acids, unsaturated OR (MM "Fatty Acids, Unsaturated+")) AND (TI (osteoporosis or bone density or bone loss) OR AB (osteoporosis or bone density or bone loss) OR TI bone mineral density OR AB bone mineral density OR TI bone health OR AB bone health OR (MM "Osteoporosis+") OR (MM "Bone and Bones+"))

Supplemental Figure S1: Subgroup analysis for gender. a, lumbar spine BMD; b, femoral neck BMD; c, CTx-1; d, NTx-1; e, BAP; f, OC; g, serum calcium; h,25(OH)D; i, PTH; j, blood n-3 PUFA.

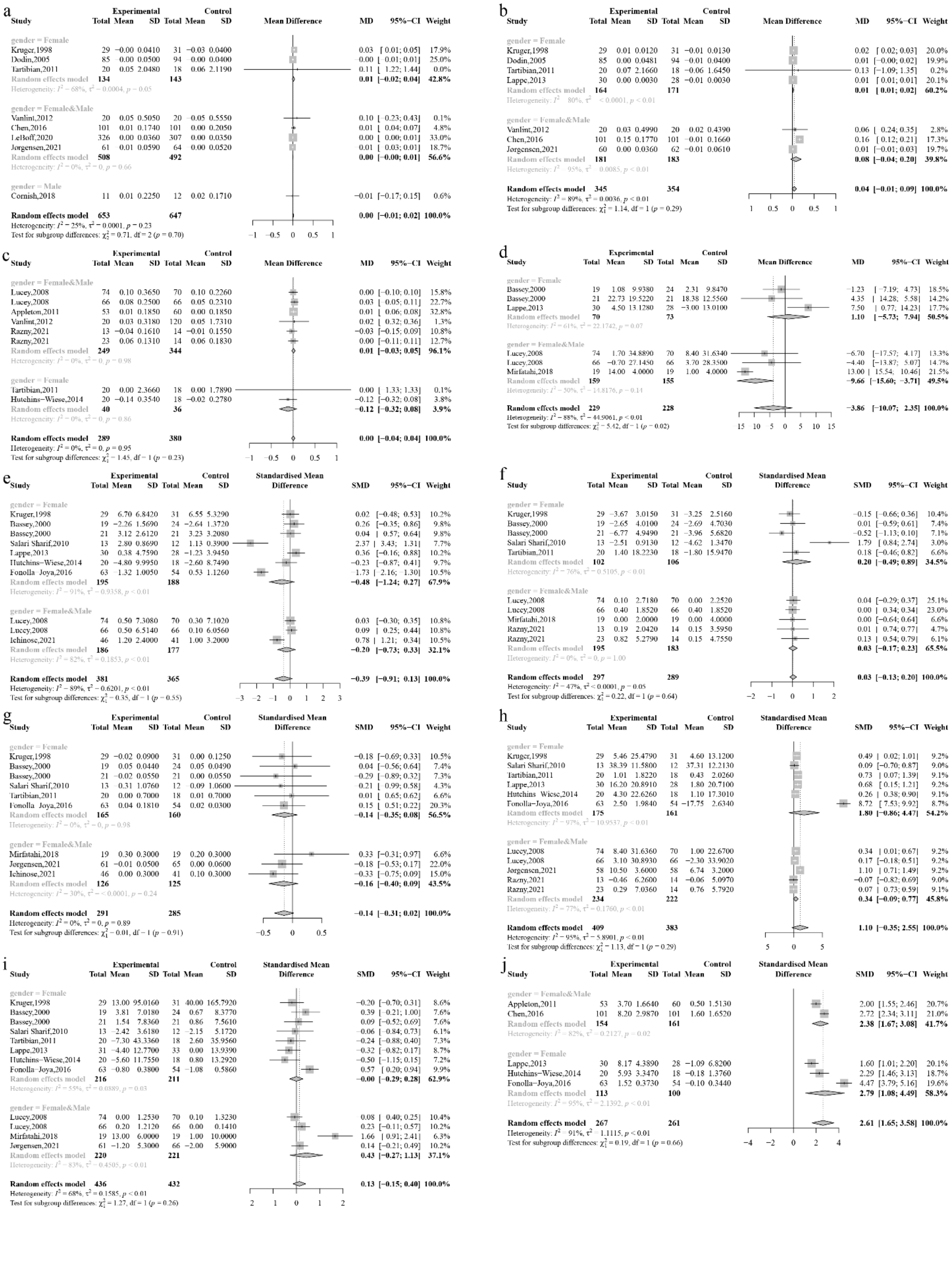

Supplemental Figure S2: Subgroup analysis for average age. a, lumbar spine BMD; b, femoral neck BMD; c, CTx-1; d, BAP; e, OC; f, serum calcium; g, 25(OH)D; h, PTH; i, IL-6; j, blood n-3 PUFA.

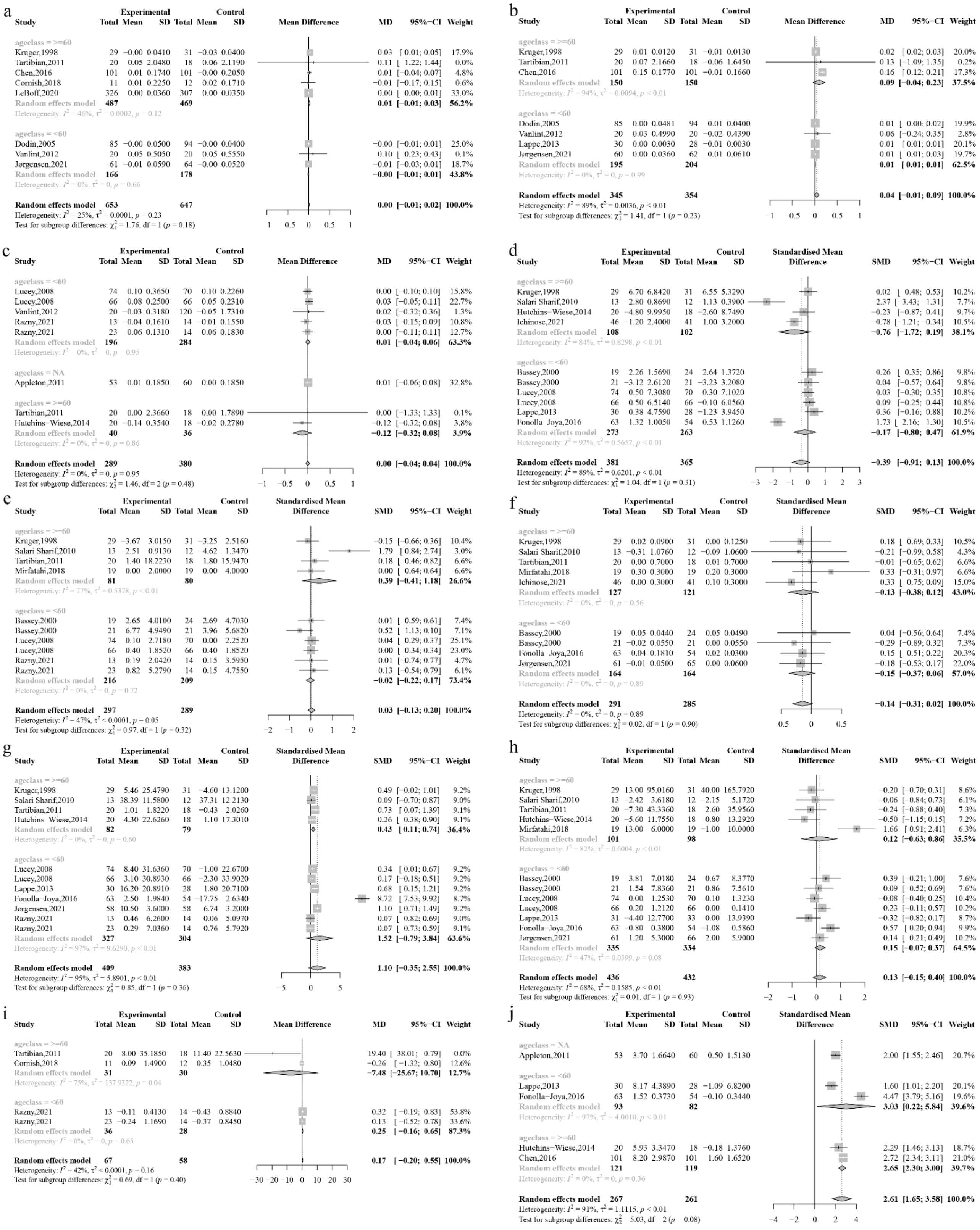

Supplemental Figure S3: Subgroup analysis for control type. a, lumbar spine BMD; b, femoral neck BMD; c, CTx-1; d, BAP; e, OC; f, serum calcium; g, 25(OH)D; h, PTH; i, IL-6; j, blood n-3 PUFA.

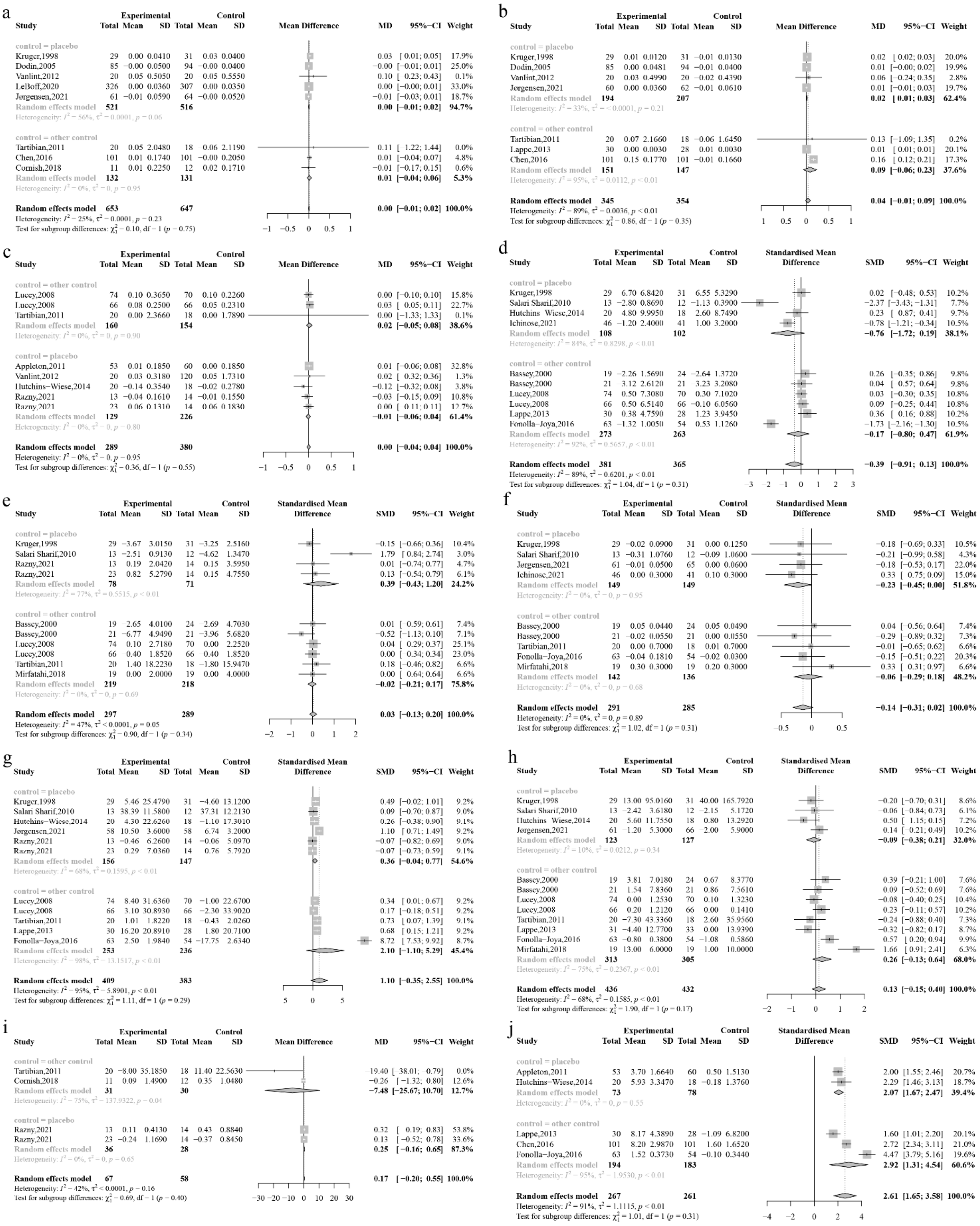

Supplemental Figure S4: Subgroup analysis for country. a, lumbar spine BMD; b, femoral neck BMD; c, CTx-1; d, BAP; e, OC; f, serum calcium; g, 25(OH)D; h, PTH.

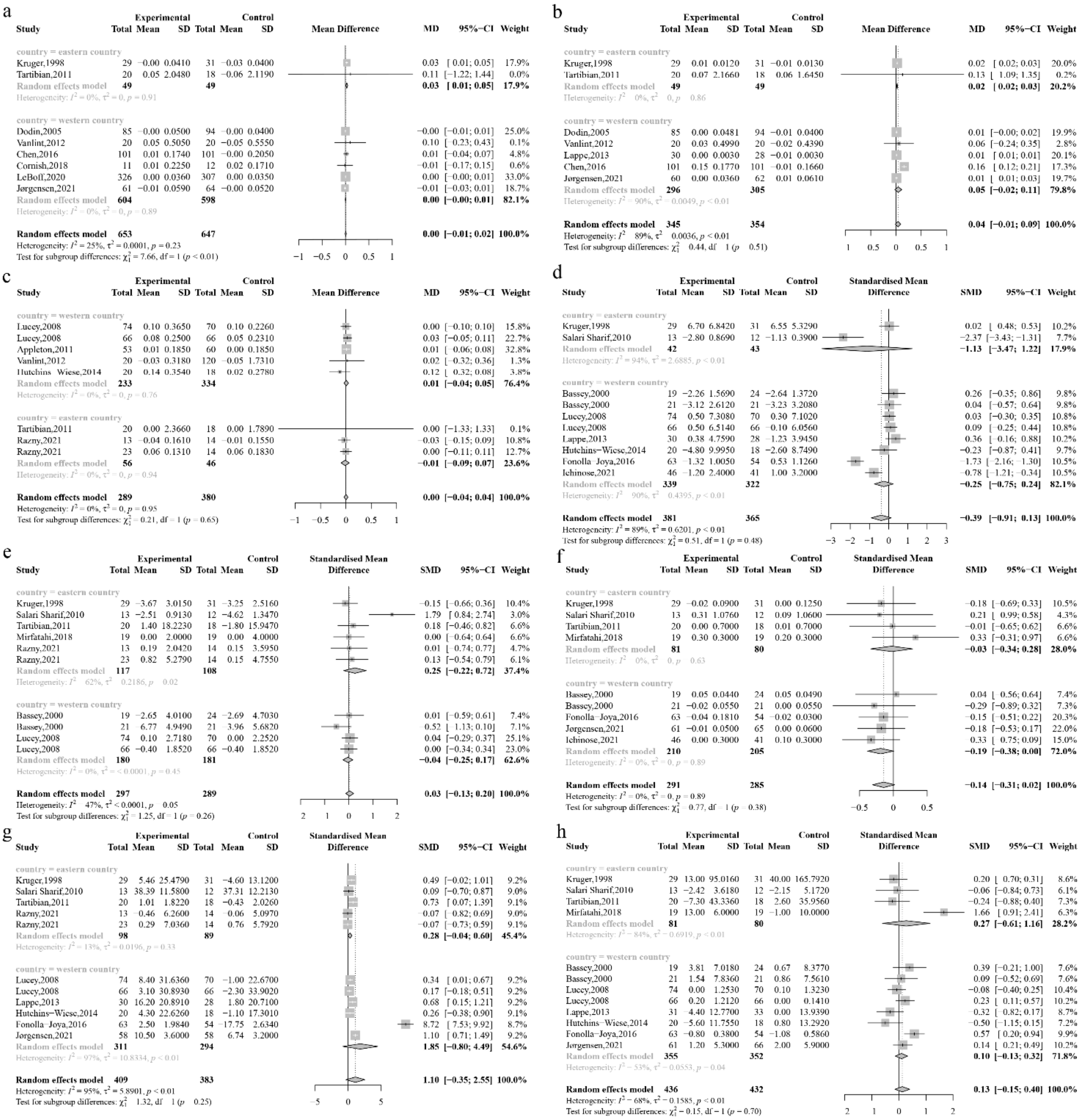

Supplemental Figure S5: Subgroup analysis for intervention type. a, TBMD; b, lumbar spine BMD; c, femoral neck BMD; d, NTx-1; e, BAP; f, OC; g, serum calcium; h, 25(OH)D; i, PTH; j, blood n-3 PUFA.

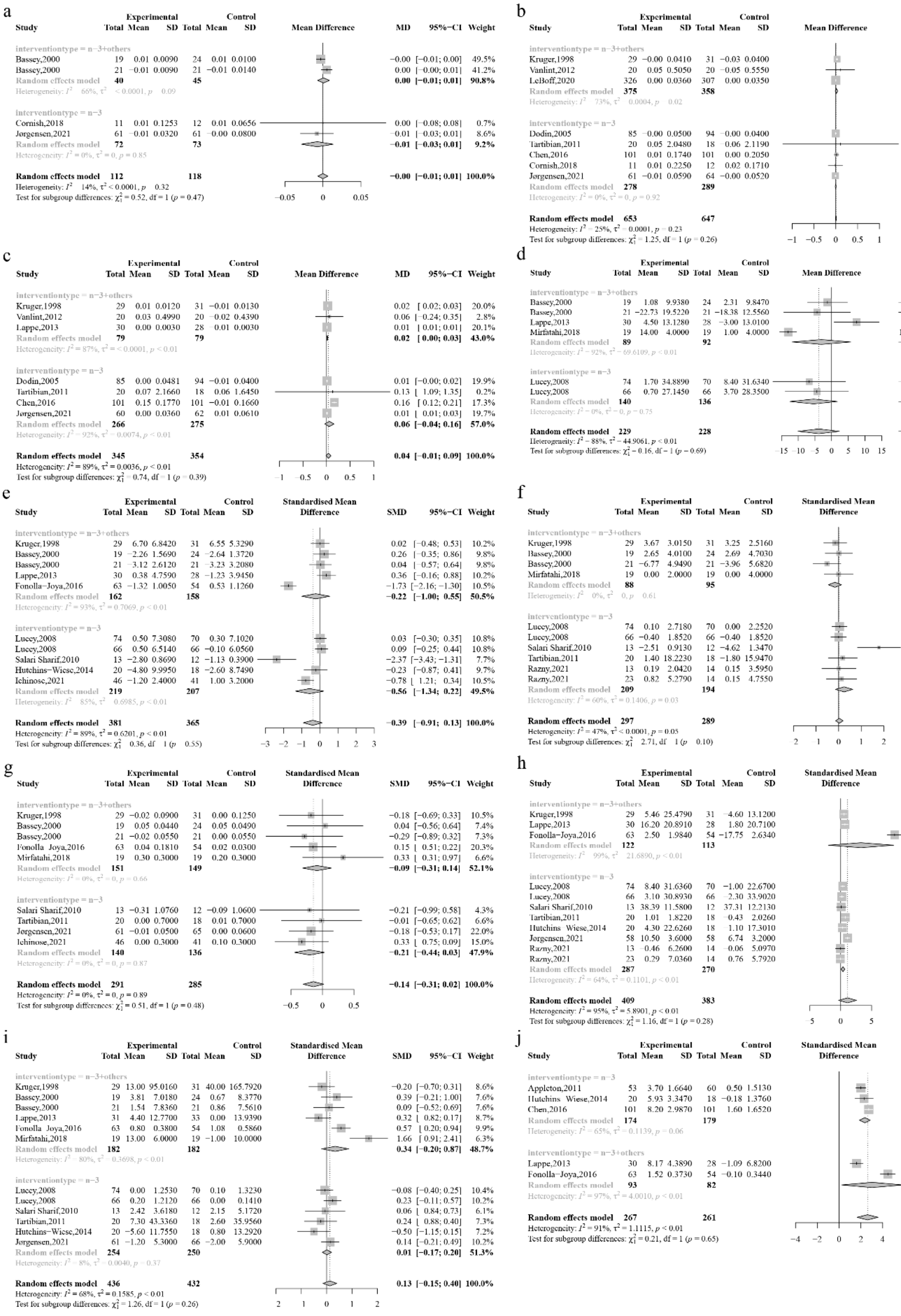

Supplemental Figure S6: Subgroup analysis for duration. a, lumbar spine BMD; b, femoral neck BMD; c, NTx-1; d, BAP; e, OC; f, serum calcium; g, 25(OH)D; h, PTH; i, blood n-3 PUFA.

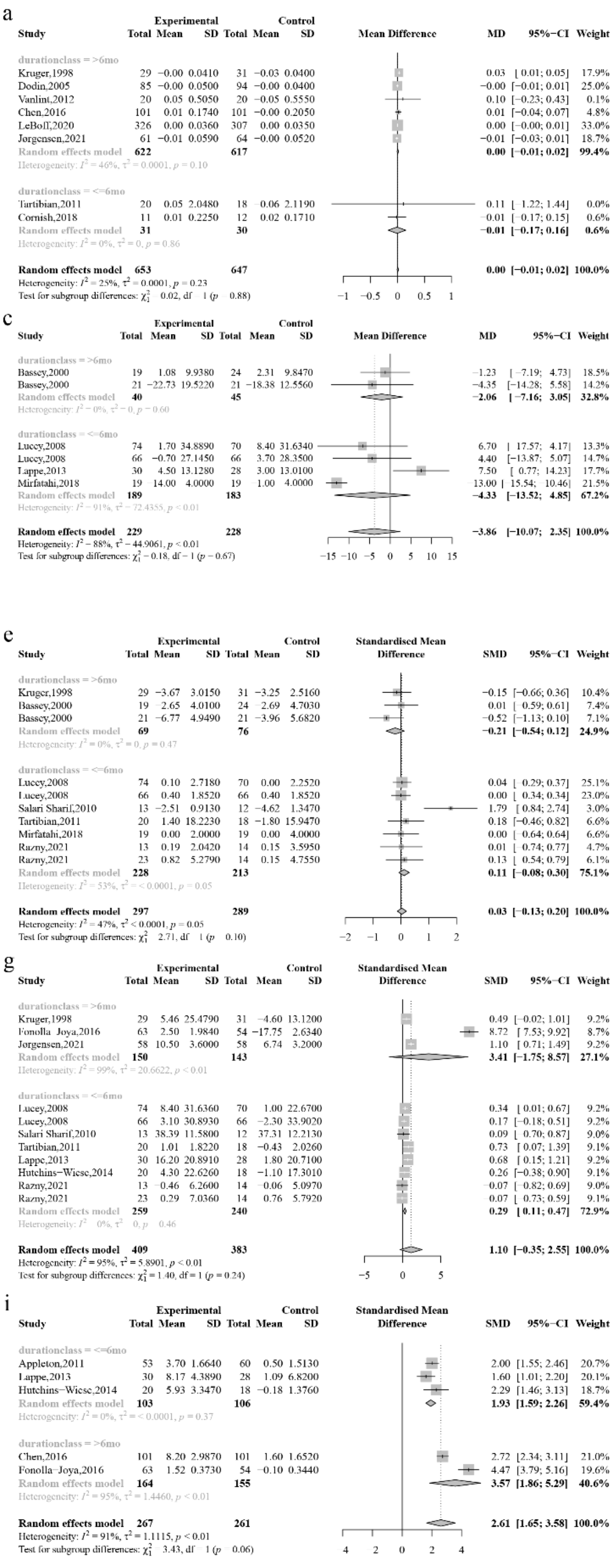

Supplemental Figure S7: Subgroup analysis for n-3 PUFAs' dose. a, TBMD; b, lumbar spine BMD; c, femoral neck BMD; d, CTx-1; e, NTx-1; f, BAP; g, OC; h, serum calcium; i, 25(OH)D; j, PTH; k, blood n-3 PUFA.

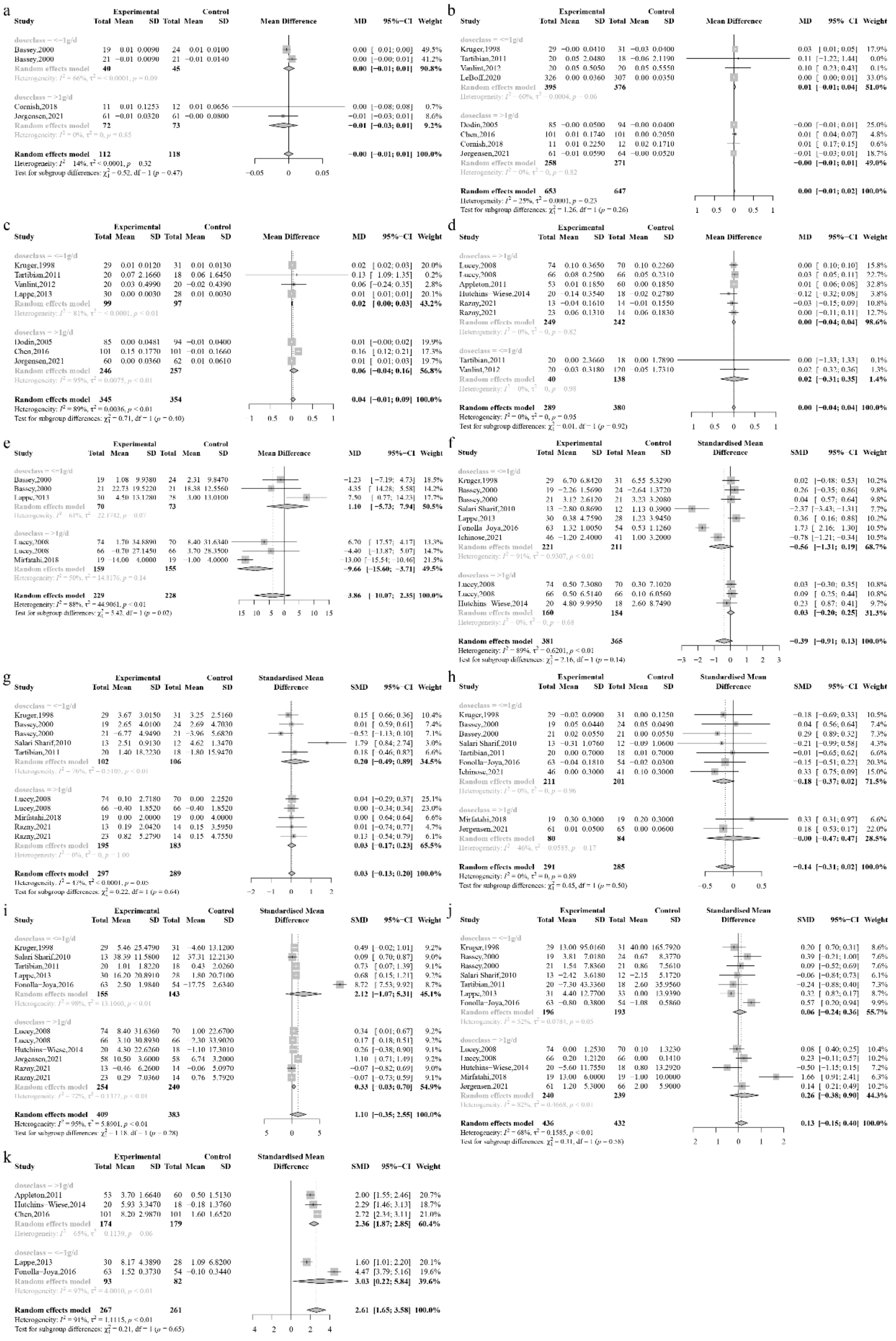

Supplemental Figure S8: Sensitivity analysis of the effects of n-3 PUFAs. a, TBMD; b, total hip BMD; c, lumbar spine BMD; d, femoral neck BMD; e, CTx-1; f, NTx-1; g, BAP; h, OC; i, serum calcium; j, 25(OH)D; k, PTH; l, blood n-3 PUFA; m, blood n-3 PUFA: n-6 PUFA ratio; n, CRP; o, IL-6.

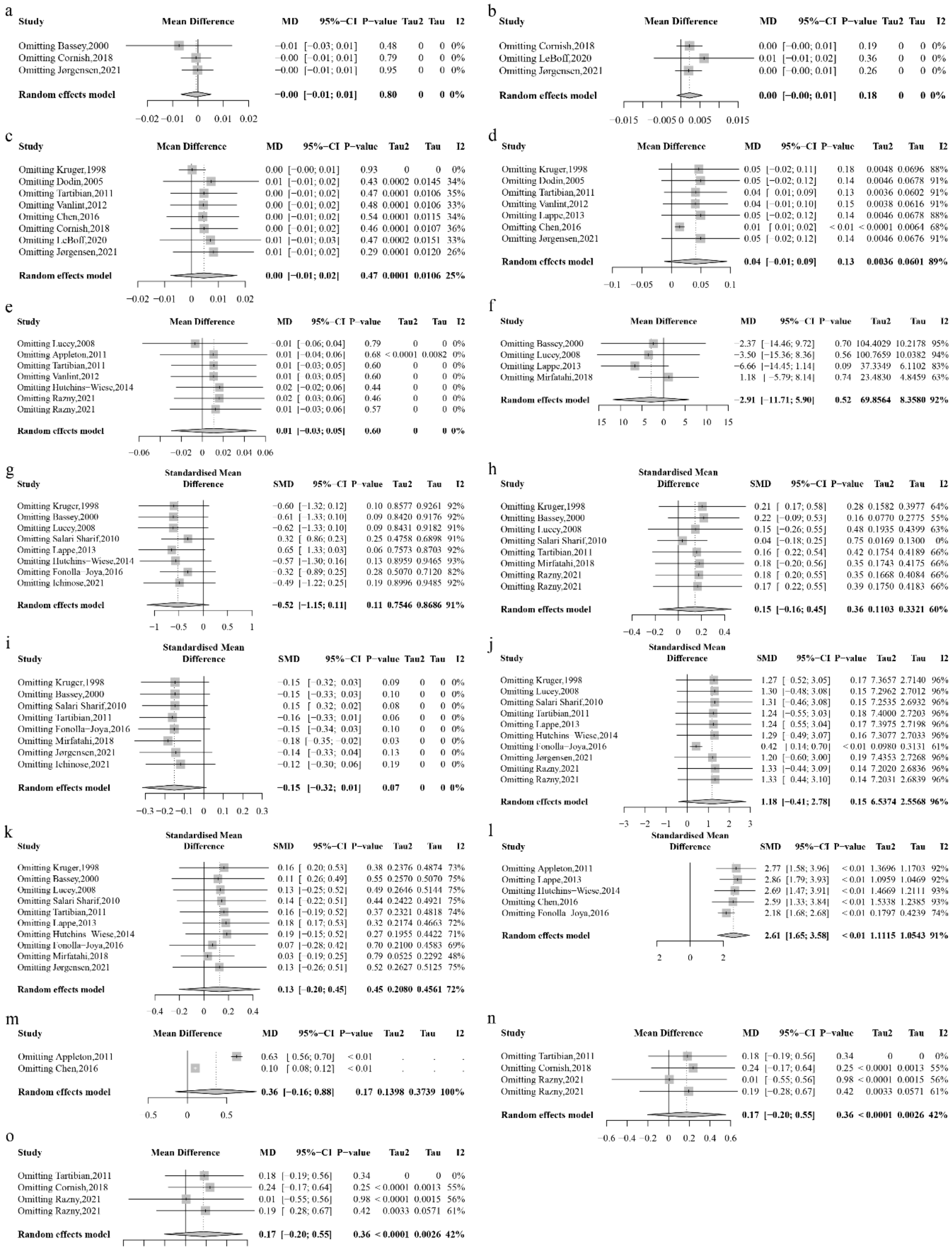

Supplemental Figure S9: Cumulative meta-analysis of the effects of n-3 PUFAs. a, TBMD; b, total hip BMD; c, lumbar spine BMD; d, femoral neck BMD; e, CTx-1; f, NTx-1; g, BAP; h, OC; i, serum calcium; j, 25(OH)D; k, PTH; l, blood n-3 PUFA; m, blood n-3 PUFA: n-6 PUFA ratio; n, CRP; o, IL-6.

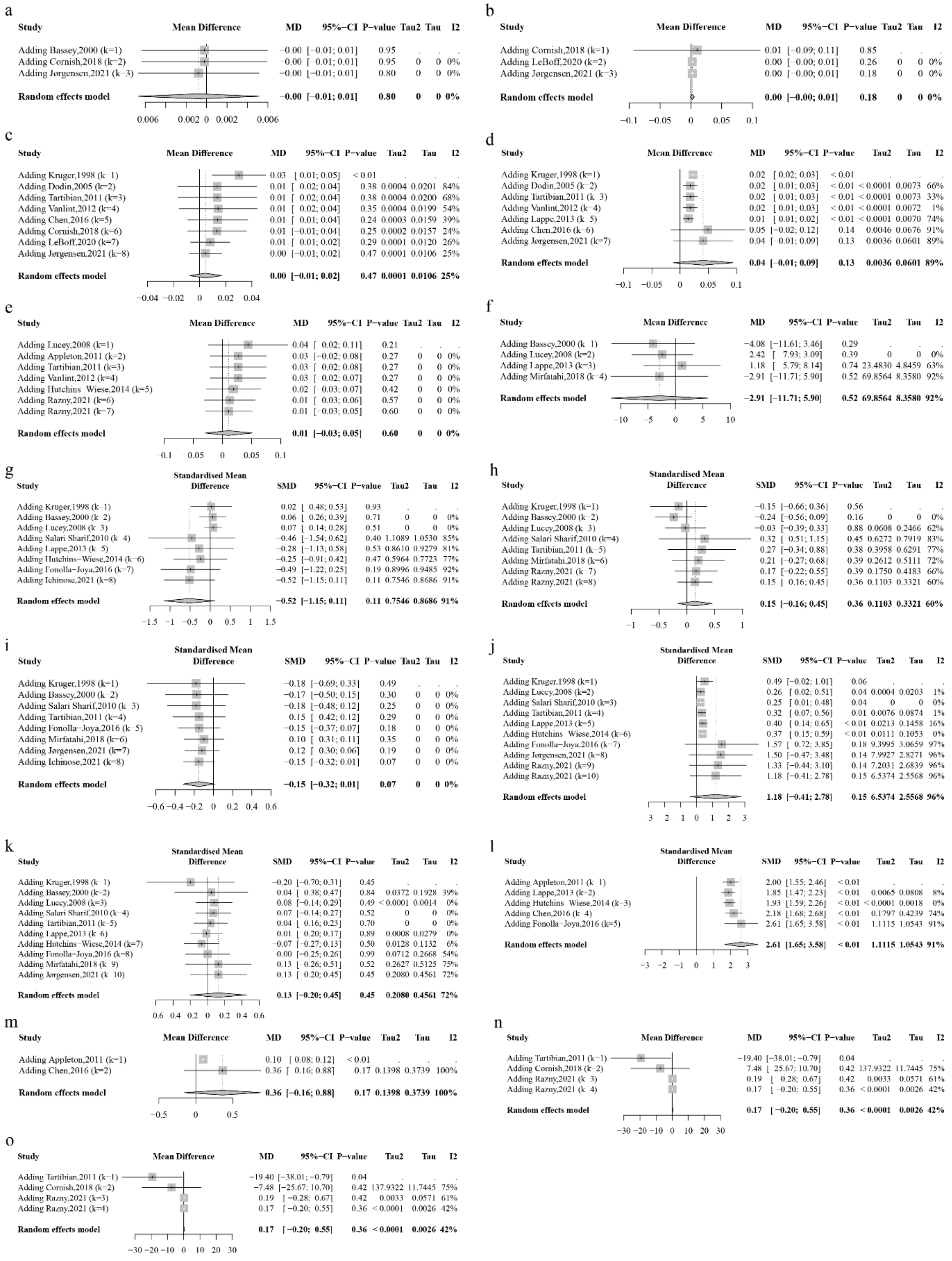

Supplemental Figure S10: Funnel plot for included studies. a, TBMD; b, lumbar spine BMD; c, total hip BMD; d, femoral neck BMD; e, CTx-1; f, NTx-1; g, BAP; h, OC; i, serum calcium; j, 25(OH)D; k, PTH; l, blood n-3 PUFA; m, blood n-3 PUFA: n-6 PUFA ratio; n, CRP; o, IL-6.

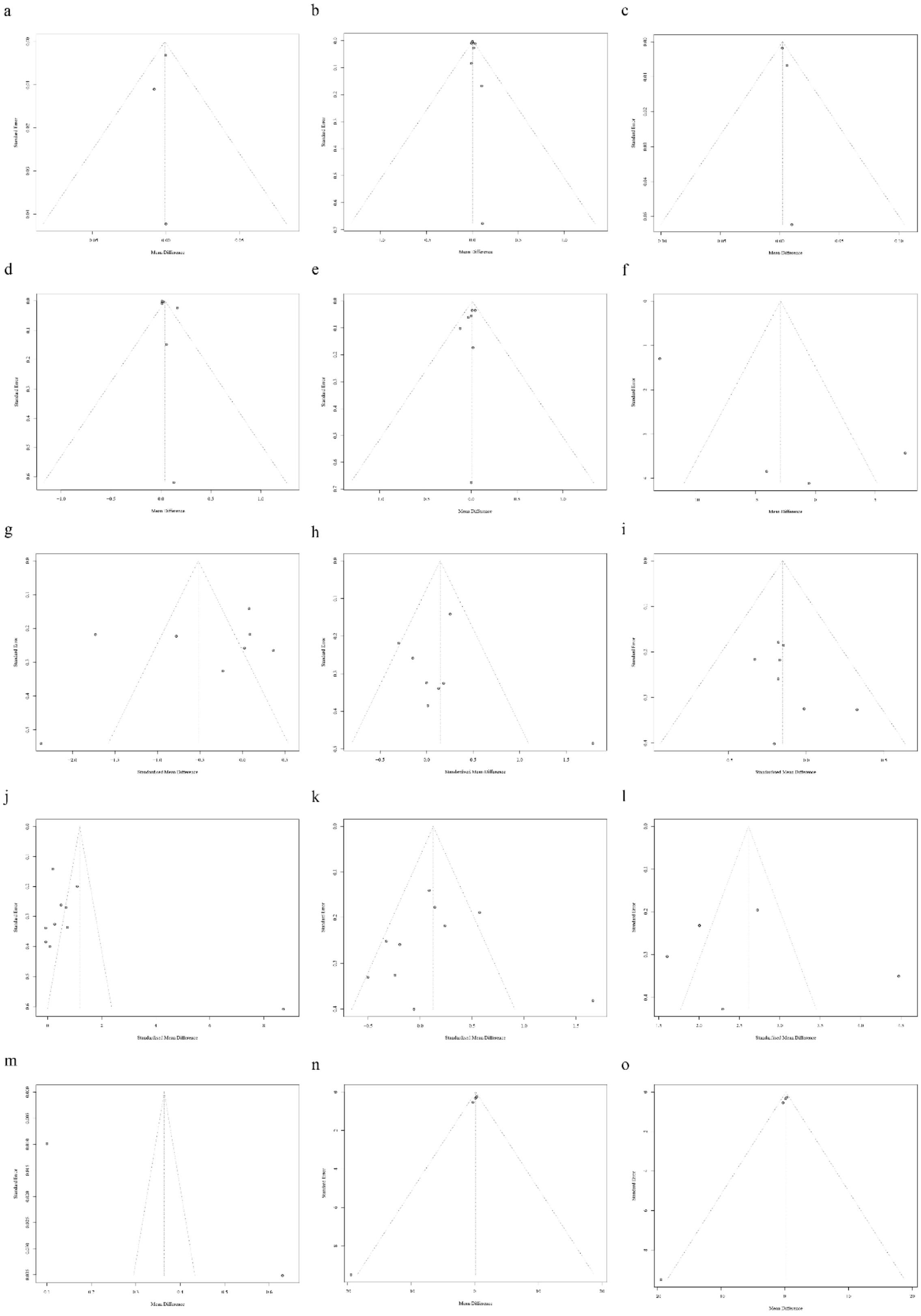

Supplemental Figure S11: Forest plot of trim-and-fill method for included studies. a, TBMD; b, total hip BMD; c, lumbar spine BMD; d, femoral neck BMD; e, CTx-1; f, NTx-1; g, BAP; h, OC; i, serum calcium; j, 25(OH)D; k, PTH; l, blood n-3 PUFA; m, CRP; n, IL-6.

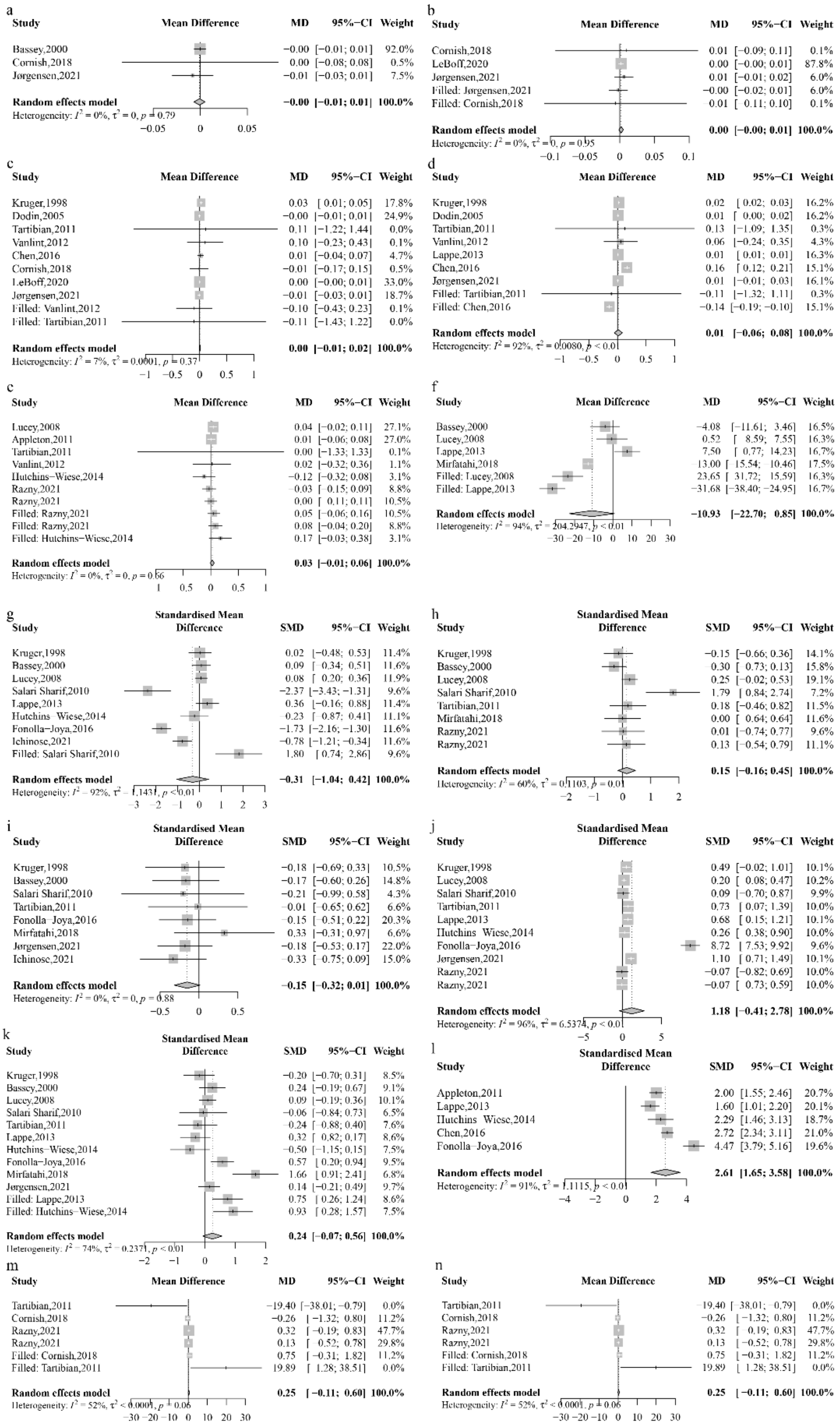

Supplemental Figure S12: Funnel plot of trim-and-fill method for included studies. a, TBMD; b, lumbar spine BMD; c, total hip BMD; d, femoral neck BMD; e, CTx-1; f, NTx-1; g, BAP; h, OC; i, serum calcium; j, 25(OH)D; k, PTH; l, blood n-3 PUFA; m, CRP; n, IL-6.

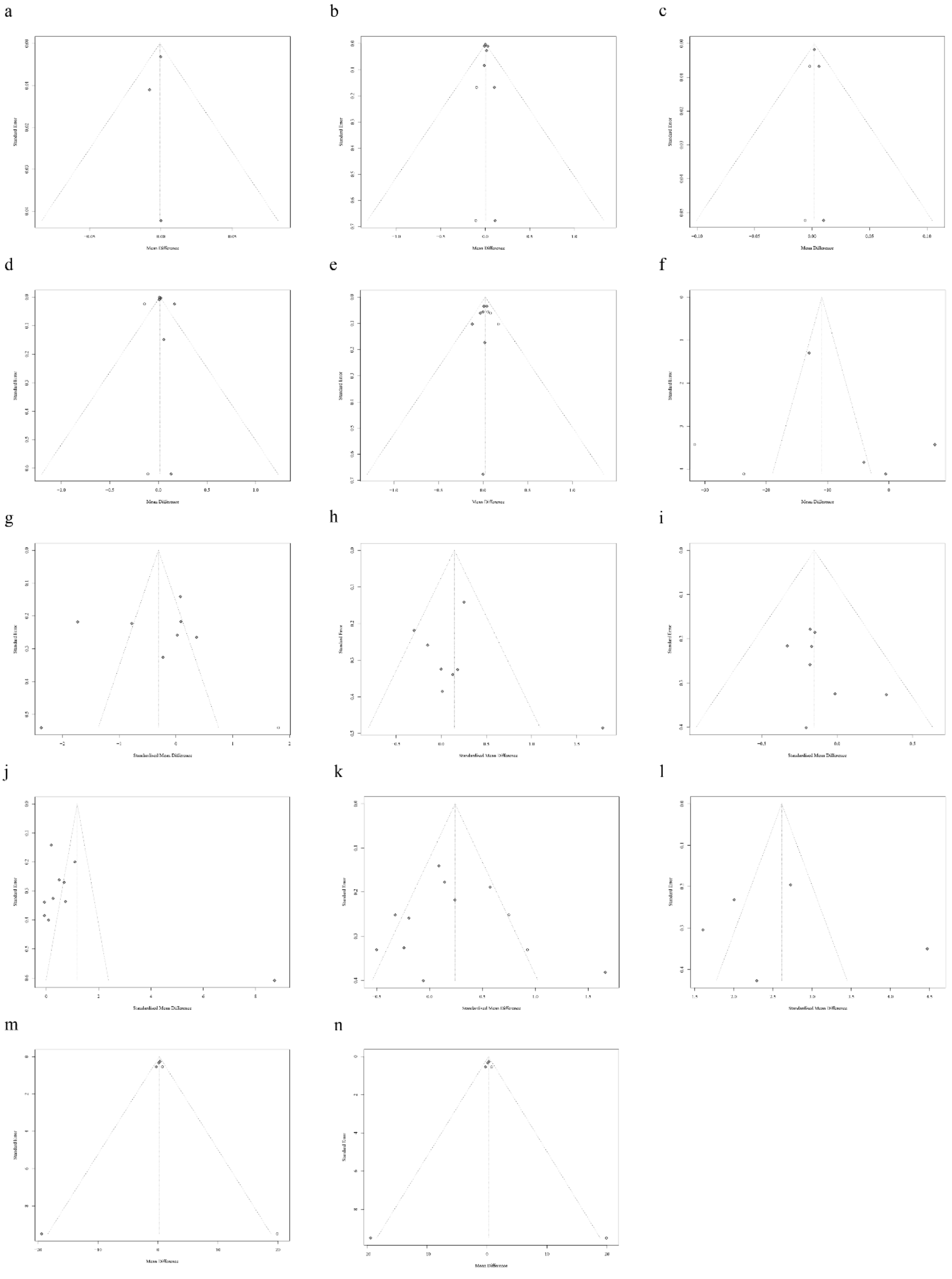

Supplement: Supplementary file 1 [file nutrients-15-02806-s001.zip › nutrients-2430773-supplementary.pdf]
